# Innervation of the clavicular part of the deltoid muscle by the lateral pectoral nerve

**DOI:** 10.1002/ca.23555

**Published:** 2020-01-13

**Authors:** Alexey Larionov, Peter Yotovski, Karl Link, Luis Filgueira

**Affiliations:** ^1^ Faculty of Science and Medicine, Anatomy University of Fribourg Fribourg Switzerland; ^2^ Faculty of Medicine University of Zurich, Institute of Anatomy Zurich Switzerland

**Keywords:** axillary nerve, brachial plexus, deltoid muscle, lateral pectoral nerve

## Abstract

**Introduction:**

The innervation pattern of the clavicular head of the deltoid muscle and its corresponding topography was investigated via cadaveric dissection in the present study, focusing on the lateral pectoral nerve.

**Materials and methods:**

Fifty‐eight upper extremities were dissected and the nerve supplies to the deltoid muscle and the variability of the lateral pectoral and axillary nerves, including their topographical patterns, were noted.

**Results:**

The clavicular portion of the deltoid muscle received a deltoid branch from the lateral pectoral nerve in 86.2% of cases. Two topographical patterns of the lateral pectoral nerve were observed, depending on the branching level from the brachial plexus: a proximal variant, where the nerve entered the pectoral region under the clavicle, and a distal variant, where the nerve entered the pectoral region from the axillary fossa around the caudal border of the pectoralis minor. These dissection findings were supported by histological confirmation of peripheral nerve tissue entering the clavicular part of the deltoid muscle.

**Conclusions:**

The topographical variations of the lateral pectoral nerve are relevant for orthopedic and trauma surgeons and neurologists. These new data could revise the interpretation of deltoid muscle atrophy and of thoracic outlet and pectoralis minor compression syndromes. They could also explain the residual anteversion function of the arm after axillary nerve injury and deficiency, which is often thought to be related to biceps brachii muscle function.

## 1 INTRODUCTION

The deltoid is a triangular multipennate muscle of the shoulder girdle. It can be characterized as plurifunctional as it participates in stabilizing the humeral articulation and provides a broad spectrum of upper limb motion. The deltoid is one of the thickest and strongest muscles of the shoulder girdle, contributing around 20% of the muscle mass of the shoulder region (De Wilde, Audenaert, Barbaix, Audenaert, & Soudan, 2002; Lee & An, 2002; Rosso et al., 2014). It originates from the lateral 2/3 of the scapular spine, the acromion, and the lateral 1/3 of the clavicle. Its insertion is the deltoid tuberosity of the humerus (Moatshe et al., 2018; Torpey et al., 1998). According to its origin, the deltoid divides into three portions: clavicular (anterior), acromial (middle), and spinal (posterior) (Moser et al., 2013). These portions can be divided into seven segments, which are associated with corresponding intramuscular tendons. The anterior head contains two segments (S1–S2), the middle a single segment (S3), and the posterior four segments (S4–S7; Audenaert & Barbaix, 2008; Axiarlis, Noussios, & Natsis, 2015; Gorelick & Brown, 2007; Leijnse, Han, & Kwon, 2008).

The deltoid belongs to a group of muscles with several lines of pull (Ackland & Pandy, 2009; Billuart, Devun, Skalli, Mitton, & Gagey, 2008). Therefore, it can provide both simple and complex motions of the upper extremity. Depending on the mobility of the arm (freely mobile or fixed), the deltoid promotes different actions: abduction, partial adduction, flexion, extension, medial rotation, and lateral rotation (Morris, 1914).

Its main function is an abduction movement. However, it can also contribute to adduction during the synergetic action of the clavicular and spinal heads. The three heads with seven segments of the deltoid can be activated independently during different phases of the various movements (Gorelick & Brown, 2007). The clavicular portion provides flexion, medial rotation, and abduction of the arm. The acromial portion provides abduction of the arm. The spinal portion is responsible for external rotation and extension of the upper extremity (Sinelnikov, 1988; Vankov & Ovcharov, 2008). According to Gorelick et al., the clavicular portion (Segments S1 and S2) represents the primary flexion mover while the segments of the spinal portion act on extension (S7) and external rotation (S4–S6). The main contributor to abduction movements is the acromial head (S3), supported by the other two heads (Gorelick & Brown, 2007). The agonistic and antagonistic relationships between the muscle parts and segments are complicated and should be viewed in relation to their actions. The antagonists for the deltoid muscle are pectoralis major and supraspinatus.

Most standard anatomy books state that the axillary nerve is the exclusive supplier of innervation to the deltoid. The topography of the axillary nerve in this muscle is well defined. There are three possible distribution patterns: (a) a single branch to the teres minor, an anterior branch to the clavicular and acromial deltoid, and a posterior branch to the spinal deltoid (32%); (b) an anterior branch to the posterior two‐thirds of the deltoid and a posterior branch to the teres minor (62%); (c) an anterior branch to the acromial and clavicular deltoid and a posterior branch to the spinal deltoid. The teres minor receives its branches from the main trunk of the axillary nerve (6%) (Stecco et al., 2010). However, the deltoid muscle can be also innervated by the lateral pectoral nerve as well as the axillary nerve (Quain, 1895). Solomon and colleagues mentioned that the deltoid can be innervated by both these nerves (Solomon, Ravindranath, Vidic, & Dym, 1997). According to Bergman's encyclopedia of anatomical variations, the deltoid muscle can be innervated by the medial pectoral nerve (Bergman, 2016). The lateral pectoral nerve passes over the axillary artery and perforates the clavipectoral fascia. Further, it follows the thoracoacromial vessels (Macchi et al., 2007). According to basic anatomy, the lateral pectoral nerve forms no branches; however, in 2005, it was reported that the cutaneous nerve to the acromial region arises from the lateral pectoral nerve (Akita et al., 2005). In general, the detailed topography of the lateral pectoral nerve for innervating the deltoid has not been well defined, and the incidence of this innervation variant has not been quantified in the literature. The purpose of this study was to identify the nerve distribution and topographic relationships of the lateral pectoral nerve innervating the deltoid muscle.

## 2 MATERIALS AND METHODS

### 2.1 Gross anatomy

The anatomy of the deltoid muscle and nerve supply were investigated in 58 upper limbs with no visible marks of trauma or surgery (29 right and 29 left) from 29 corpses (13 males and 16 females), which were used in gross anatomy dissection courses at the University of Fribourg. All cadavers were embalmed with Jores solution. Their ages ranged from 68 to 101 years (mean 84 ± 10 years). Exclusion criteria were surgical intervention in the clavipectoral triangle and severe trauma in the shoulder region. Classical dissection methods were used. In the first step, the skin was removed along the longitudinal axis of the clavicle, acromion, and humerus. The pectoral fascia and the clavipectoral fascia were sequentially dissected and excised. The pectoralis major and minor muscles were cautiously detached from their origins. The arms were then removed from the trunk by disarticulation of scapulothoracic and sternoclavicular joints at the middle clavicular level. Finally, the deltoid muscle was precisely separated from the clavicle, acromion, and scapular insertions. Its three portions were clearly identified. The broad nerve network, including the brachial plexus, the axillary nerve and its branches, and several branches of the lateral pectoral nerve, were identified during the various dissection steps and kept so that the patterns of the deltoid muscle innervation could be studied in detail. The dissection was photodocumented.

### 2.2 Histology

At the end of the dissection, the nerves identified were processed for histological examination. They were excised together with the innervated muscle and kept in 4% formalin before embedding. The approximate size of the biopsy sample (part of the nerve with innervated muscle) was 1.5 cm × 1.5 cm. The specimens were embedded in paraffin and 3‐μm‐thick sections were stained with Mason trichrome. Imaging and documentation were completed with a digital pathology slide‐scanner (Hamamatsu Nano Zoomer HT + Fluo).

### 2.3 Statistical analysis

The data gathered during the gross anatomy dissection from the 58 limbs were analyzed using the GraphPad Prism 8 software.

## 3 RESULTS

**Figure 1:**
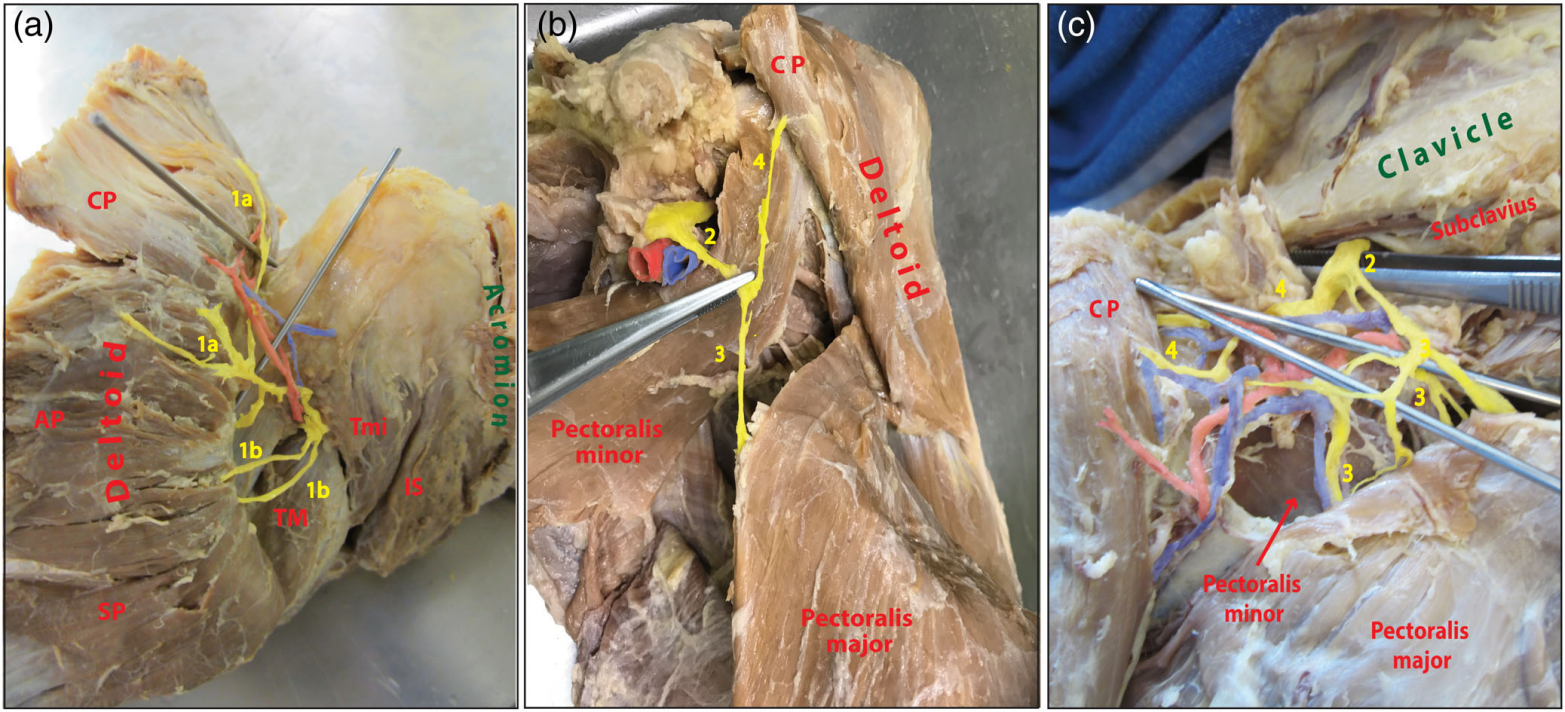
Nerve distribution patterns of the deltoid muscle. (a) “Classical” nerve distribution pattern: all portions of the deltoid muscle (clavicular, acromial, spinal) receive branches from the axillary nerve. AP, acromial part of deltoid; CP, clavicular part of deltoid; Is, infraspinatus; SP, spinal part of the deltoid; T; TM, teres major; Tmi, teresminor; 1a, anterior branches of the axillary nerve, 1b, posterior branches of the axillary nerve. (b)“Non‐classical” nerve distribution pattern: distal splitting of the lateral pectoral nerve from the brachial plexus. CP, clavicular part of deltoid; 2, lateral pectoral nerve; 3, pectoral branch of the lateral pectoral nerve; 4, deltoid branch of the lateral pectoral nerve. (c) “Non‐classical” nerve distribution pattern: proximal splitting of the lateral pectoral nerve from the brachial plexus. CP, clavicular part of deltoid; 2, lateral pectoral nerve; 3, pectoral branch of the lateral pectoral nerve; 4, deltoid branch of the lateral pectoral nerve [Color figure can be viewed at wileyonlinelibrary.com]

Anatomical dissection of 58 upper limbs (29 rights and 29 lefts) from 29 cadavers was used to investigate the relationships of the lateral pectoral nerve to the deltoid muscle and identify the probable topographic variants of the innervation. The study revealed two innervation patterns of the deltoid: by the axillary nerve or by a combination of the auxillary and the lateral pectoral nerves. The “classical” innervation pattern through the axillary nerve and its distribution in the deltoid muscle have been well defined by Stecco, Sakoma, Wysiadecki, Leechavengvongs and their colleagues (Leechavengvongs et al., 2015; Sakoma et al., 2011; Stecco et al., 2010; Wysiadecki et al., 2014). During our dissections, we confirmed the findings of those authors: the axillary nerve crossed the quadrangular space and sent anterior and posterior branches to three anatomical parts of the deltoid; the anterior branch supplied the clavicular and acromial portions, while the spinal head received innervation from the posterior branch (Figure 1a). In those cases, the axillary nerve originated from the posterior cord of the brachial plexus. However, the “classical” pattern, where the axillary nerve provided the only innervation to the deltoid, was observed in only 13.8% of cases (8/58).

In the remaining 86.2%, the axillary nerve sent no branches to the clavicular portion of the deltoid. This portion received branches from the lateral pectoral nerve. We defined this topographic variation as the “non‐classical” pattern. The lateral pectoral nerve originated from the lateral cord of the brachial plexus (Figure 1b,c). As mentioned earlier, the most frequent finding was that the lateral pectoral nerve was located in the clavipectoral triangle between the pectoralis major and pectoralis minor. However, its topography was variable in this region. According to our observations, the separation of the lateral pectoral nerve from the lateral cord could be proximal or distal to the pectoralis minor muscle (Table 1). There was distal splitting at the level of the pectoralis minor in 63.8% of all cases (37/58). The lateral pectoral nerve split into two branches, deltoid, and pectoral. The deltoid branch traveled directly to the clavicular portion of the muscle. The pectoral branches went to the pectoralis major and minor muscles. Topographically, the lateral pectoral nerve crossed the pectoralis minor parallel to the coracoid process, and about 2–6 cm from the clavicle it divided into the pectoral and deltoid branches (Figure 1b). Proximal splitting was observed in 22.4% of all dissections (13/58). In this variation of the “non‐classical” pattern, the branches of the lateral pectoral nerve had already begun to divide in the infraclavicular indentation (infraclavicular fossa). The nerve sent numerous branches to the pectoralis major and minor muscles, and a distinct deltoid branch to the medial half of the clavicular portion of the deltoid (Figure 1c).

**Table 1:** Distribution of the innervation patterns of the deltoid muscle by axillary and lateral pectoral nerves

	Axillary nerve—“classical” pattern	The lateral pectoral nerve—“non‐classical” pattern/proximal splitting	The lateral pectoral nerve—“non‐classical” pattern/distal splitting
Number of cadavers	8	13	37
Percentage	13.8%	22.4%	63.8%

In both patterns, the deltoid and pectoral branches created a broad communication network in the clavipectoral triangle that was closely associated with the thoracoacromial artery, which supplies blood to the region.

**Figure 2:**
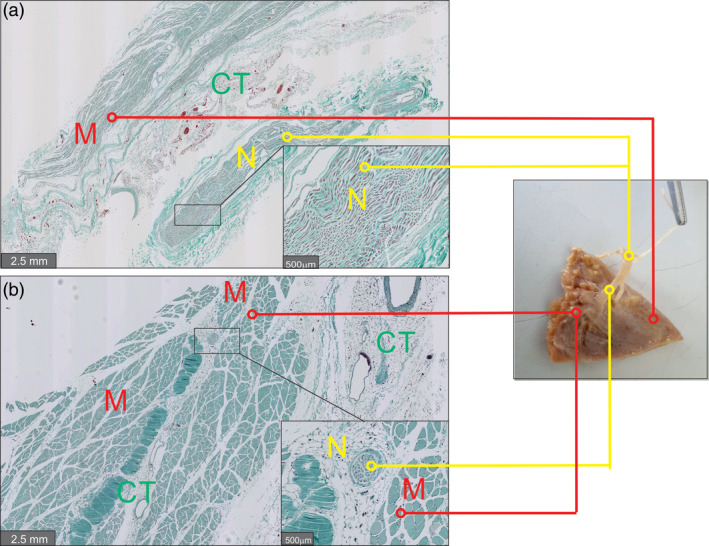
Histology of the anatomical structures. Histological assessment of the gross anatomy structures. Histological examination of biopsies from the cadavers revealed in the longitudinal shear (a) and transverse (b) sections nervous tissue with Schwann cell nuclei, axons, and myelin sheaths between the muscle fibers. CT, connective tissue, M, muscle; N, nerve [Color figure can be viewed at wileyonlinelibrary.com]

Histological examination of the deltoid nerve branches as they entered the clavicular deltoid muscle confirmed that the dissected structures were nerves accompanied by corresponding blood vessels (Figure 2).

## 4 DISCUSSION

The results of this anatomical study demonstrate that in most cases the deltoid muscle has a double nerve supply: (a) from the axillary nerve and (b) from the lateral pectoral nerve. The topography of the latter is more complex than stated in most anatomy textbooks. According to our findings, the lateral pectoral nerve divides into deltoid and pectoral branches, which carry innervation to the deltoid and pectoral muscles. The clinical significance of the present work is substantial. These data are important not only for trauma and orthopedic surgery, but also for classical neurology.

The current outcomes are consistent with the topographic investigation of the axillary nerve by Stecco and colleagues, who pointed out that the anterosuperior quadrant of the deltoid muscle (which corresponds to clavicular part of the deltoid) has no axillary nerve branches. This quadrant is a safe place for the axillary nerve during the surgical approach (Stecco et al., 2010). Indeed, in the “non‐classical” pattern of nerve distribution, we found no macroscopic branches to the clavicular portion from the axillary nerve. Physiologically separate stimulations of the axillary and the lateral pectoral nerves could confirm our findings, although this would be technically difficult.

The topographic anatomical innervation patterns described would also explain unclear published cases. In 2012, a group of neurologists published a case report about “painful shoulder‐moving deltoid syndrome.” They described a patient with severe shoulder syndrome and involuntary deltoid contraction. The pain was treated with prescription medication and surgical intervention (anterior cervical discectomy with C4–C5, C5–C6, and C6–C7 fusions). However, the muscle contraction was localized mainly in the posterior and middle portions of the deltoid (Mari, Darwin, & Hallett, 2012). This pathological condition was not explained, though it was concluded that peripheral paresis could have been implicated. In line with the present results, it was probably a typical axillary nerve dysfunction provoked by different causes (concomitant diseases or traumatic, nontraumatic, and/or idiopathic conditions). Targeted treatment of the axillary nerve could have helped to resolve the problem without surgical intervention.

During the last decade, clinical cases concerning atrophy of the deltoid muscle have been published. Authors have described the arduous atrophy of the deltoid muscle with stiff pain syndrome but no history of trauma (Monteleone, Gismant, Stevanato, & Tiloca, 2015; Weber, Burnett, & Kortebein, 2013). All publications have pointed out the role of the axillary nerve in initiating the muscle atrophy. However, the axillary nerve alone is not necessarily responsible for all atrophic conditions. The lateral pectoral nerve could be implicated in the pathological process and could provoke, for example, isolated atrophy of the clavicular portion of the deltoid muscle with intense pain and shoulder joint dysfunction. The clavicular orthopedic is vital for movements such as flexion, medial rotation, and abduction (Klepps et al., 2004; Longo, Petrillo, Rizzello, Candela, & Denaro, 2016). On the basis of the topography of the lateral pectoral nerve described here, repetitive spike movements could easily initiate nerve injury. Recently, this assumption has become a topical issue for sportsmen (basketball, volleyball, baseball players) and for people who have to use spiking movements for extended periods related to their work (plasterers, house painters, decorators). Failure of the deltoid and pectoral branches of the lateral pectoral nerve can incite isolated or reciprocal dysfunction of the deltoid and pectoral muscles. These details should be considered by neurologists for excluding peripheral paresis.

Thoracic outlet and pectoralis minor compression syndromes are also related to lateral pectoral nerve topography. These pathological conditions could be provoked by compression of the lateral pectoral nerve and surrounding vessels. Many external factors can cause compression of the neurovascular bundle (Wang et al., 2019). Squeezing of the nerves and vessels in the thoracic outlet is referred to as thoracic outlet syndrome. The thoracic outlet is divided into three areas: (a) scalene triangle (C5–C8 and T1 roots, subclavian artery); (b) costoclavicular space (the space between the clavicle, the first rib with trunks of the brachial plexus and the subclavian artery and vein); (c) pectoralis minor space (space with three trunks, cords of the brachial plexus and a vascular bundle; Sul et al., 2019). Pectoralis minor compression syndrome is a constriction of the elements of the sub‐pectoral tunnel (Ammi et al., 2018). The main clinical signs of these two pathologies are almost identical and include pain in the shoulder, paraesthesia, numbness of the upper extremity, and weakness of the upper limb. However, intense pain in the anterior part of the chest wall, and pain when the pectoralis minor is palpated below the coracoid process, are typical symptoms of pectoralis minor compression syndrome.

The proximal variation of the pectoral nerve, mentioned in this article, was previously described by Shetty and colleagues in 2014. They found that the lateral pectoral nerve originates from the upper trunk of the brachial plexus and crosses under the clavicle into the clavipectoral triangle (Shetty, Nayak, Kumar, Thangarajan, & D'Souza, 2014). We did not observe this topographical variant of the lateral pectoral nerve splitting from the superior trunk of the brachial plexus, so this pattern can be regarded as an individual isolated variation. On the other hand, the topographical projection of the lateral pectoral nerve in our cases followed the direction of the midclavicular line, in agreement with Shetty and colleagues.

**Figure 3:**
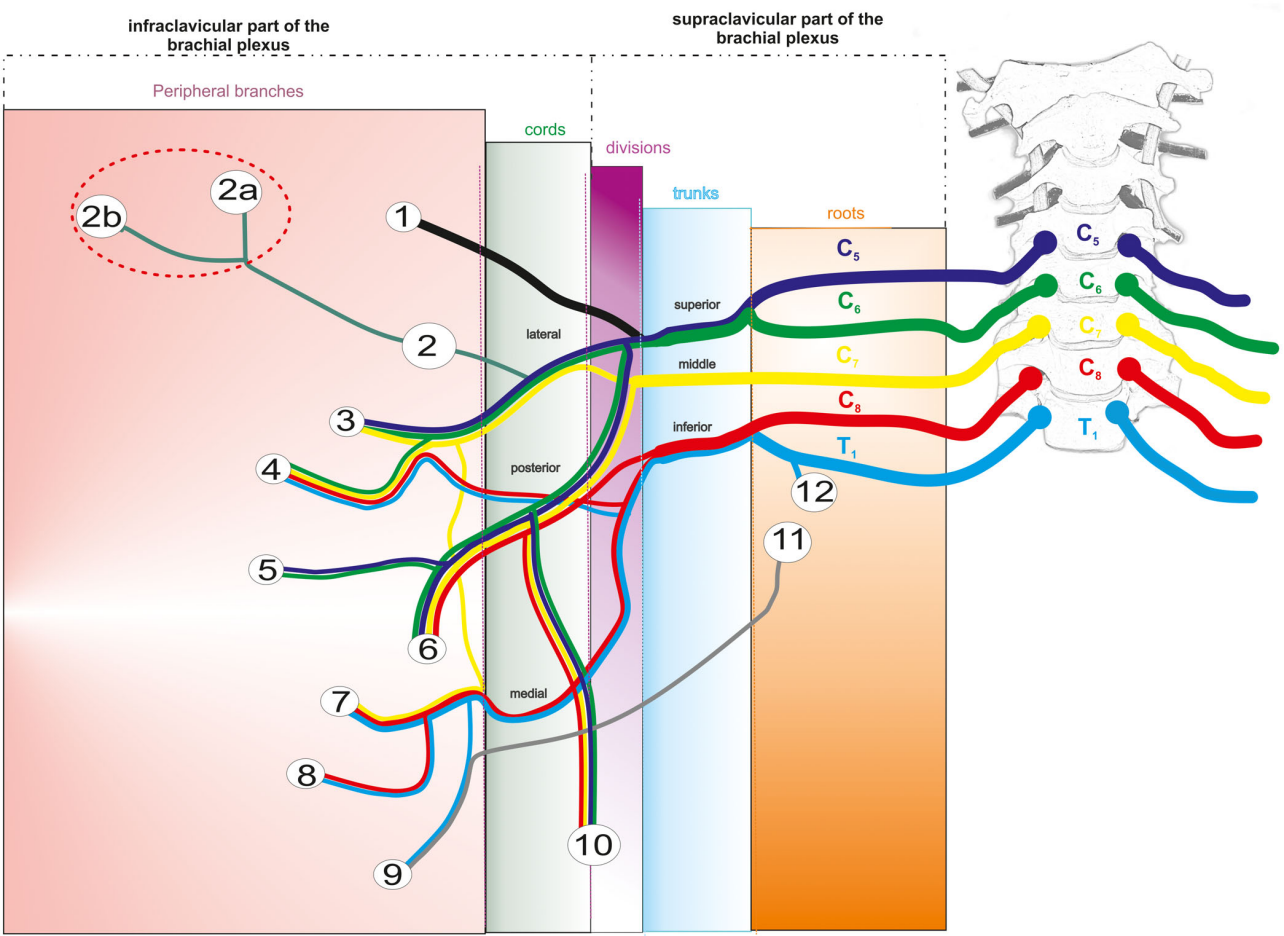
Scheme of the brachial plexus and the terminal branches of the lateral pectoral nerve. 1, suprascapular nerve; 2, lateral pectoral nerve; 2a, deltoid branches of the lateral pectoral nerve; 2b, pectoral branches of the lateral pectoral nerve; 3, musculocutaneous nerve; 4, median nerve; 5, axillary nerve; 6, radial nerve; 7, ulnar nerve; 8, medial cutaneous nerve of forearm; 9, medial cutaneous nerve of arm; 10, thoracodorsal nerve; 11–12, intercostobrachial nerves [Color figure can be viewed at wileyonlinelibrary.com]

The distal splitting of the lateral pectoral nerve is described for the first time in our study. In this paradigm, the lateral pectoral nerve originates directly from the lateral brachial cord, comes out below the clavicle and separates into its final branches, pectoral and deltoid (Figure 3).

In conclusion, we have characterized the topographic features of the lateral pectoral nerve, classified its different topographic variations, and demonstrated a double nerve supply to the deltoid muscle that accords with previous publications. Our findings are essential for orthopedic surgeons and neurologists, who evaluate patients with shoulder dysfunction daily and treat them routinely.
